# Factors That Guide the Diagnosis and Treatment Planning for Impacted Canines Using Three-Dimensional Cone-Beam Computed Tomography: A Cross-Sectional Study

**DOI:** 10.1155/2022/7582449

**Published:** 2022-10-03

**Authors:** Hasan Sabah Hasan, Mohamed A. Elkolaly, Ramy Elmoazen, Ayshan Kolemen, Arkan Muslim Al Azzawi

**Affiliations:** ^1^Orthodontic Department, Azadi Dental Teaching Center, General Directorate of Hawler, Ministry of Health, Erbil, Iraq; ^2^Orthodontic Department, Privet Specialized Dental Clinic, Hawler New, Erbil, Iraq; ^3^Orthodontic Department, Royal Dental Center, Alexandria, Egypt; ^4^School of Computing, University of Eastern Finland, Joensuu 80100, Finland; ^5^Orthodontic Department, Al-Mustaqbal University College Dentistry, Babil, Iraq; ^6^Orthodontics Department, College of Dentistry-university of Babylon, Babil, Iraq

## Abstract

**Objective:**

Impacted canines are one of the significant challenges in orthodontics that should be appropriately assessed to provide the best treatment to the patients.

**Materials and Methods:**

In the present study, 57800 patients were examined over six years to investigate the prevalence, diagnostic procedures, and treatment methodologies for impacted canine cases. Prevalence and diagnosis were tested using history taking, clinical examination, and three-dimensional cone-beam radiographs. The cases were tested for impaction site, gender, age, signs, and symptoms. The groups were classified for impaction location according to Mupparapu's classification.

**Result:**

The causative factors and the treatment methodology selected were plotted according to age and gender distribution. The total prevalence was 3.9% of canine impaction cases in relation to the total sample cases. The results showed a strong correlation between the site of impaction toward the upper arch and with distribution following Mupparapu's classification. The pain was the most detectable complication in all age groups, while root resorption was the least.

**Conclusion:**

Most of the younger age groups were sent for exposure and orthodontic traction, while the mid-aged groups elected for observation, and follow-up as their primary concern was esthetics. However, the adult patients were into exposure and traction to improve their function.

## 1. Introduction

Impacted canine cases were part of the fundamental challenges in the orthodontic field since the evolvement of orthodontic treatment. Nevertheless, canine impaction cases were frequent. Almost every orthodontist treated at least one case of that category [1]. An impacted canine, by definition, is a canine with completed root formation and prevented from eruption into position by another tooth, bone, soft tissue, or pathology [2]. Hypodontia in canines was considered very rare, with a total incidence of 0.08%, knowing that one should always consider the canine to be impacted or extracted if not found intraorally [3]. Impacted canines, on the other hand, were represented by an incidence of 2% [4, 5]. That ratio was considered high if you compare it to other ratios of different anomalies. If a canine was impacted, mostly it would be found palatally by 61% chance. The next probability was to be impacted in the line of the arch by 34%. Last and least, it would be found impacted buccally (4.5%). That incidence was presented more in females than males by a ratio of 2 : 1. It had been recorded that impacted canine cases were more frequent within class II cases and that distribution was more in a unilateral pattern 4 : 1 [6–8].

The results of impacted canines were dramatic and complicated, unfortunately. Knowing that the canine had the tallest root in the arch and its crown had a pointed and well-developed cusp, one could imagine the results of touch between it and other teeth. If the crown approached a root (especially the lateral incisor root), dramatic external root resorption was anticipated [9]. In the past, using two-dimensional (2D) radiographs only, a 12.5% chance of root resorption was reported. Nowadays, using three-dimensional (3D) radiographs, that incidence reached 45% [10, 11]. Even if the canine did not collide with the roots and slipped its way in between, the result would be a typical case of transposition [12]. Canines were reported to be transposed between the lateral and central incisors and between the first and second premolars. Transposition was one of the obvious problems in orthodontics, and in most cases, the clinician had to accept it and compromise the occlusion. The third possibility was that the canine chose to halt eruption and got stuck in the palatal or buccal bony plates. They were dealing with a situation that required different approaches to interdisciplinary treatment. Combined surgical and orthodontic approaches together with restorative approaches were necessary to solve such problems [13].

Different papers were published trying to investigate the etiology of the problem. Why was that dreadful fate faced by some canines only? Some suggested a genetic theory, where similar impacted scenarios were faced in families and in-between siblings [14–16]. Many cases of palatally impacted canines were reported between mothers, daughters, sisters, and cousins. Many of them presented with bilaterally impacted canines' situations, suggesting a strong genetic phenomenon.

Other authors suggested the guidance phenomenon, considering the long path of eruption and a higher chance of “getting lost” throughout the eruption path [17]. Canines develop very high beside the nasal bridge and slide downward/outward following the path of the nasolacrimal duct. That happened because of the pyramidal shape of the maxilla, with its outer surface flaring outward while descending to its lower levels [18, 19]. Canines continued to slide in such a downward/outward direction towards the buccal plate of the bone covering the teeth. When the canines approached the apices of the lateral incisors, they changed course and started to adopt a vertical eruption path, followed and slid on the roots of the lateral incisors till they reached the occlusal plane [20], knowing that the clinician could understand and predict the sequel of a missing lateral incisor or a short rooted/dilacerated/tilted/stunted incisor. Losing the well-formed lateral incisor root leads to buccal impaction of the canines. Hence, one could understand why most of the buccally impacted canines were unilateral; that was because faults of the incisors were mostly unilateral, not bilateral [21].

A third theory was that crowding and space deficiency were the main causes of canines impacted in the line of the arch. Canines could travel the eruption path correctly and slide over with the apices of the lateral incisors but stuck between the roots of incisors and premolars due to space deficiency. This explained why most cases of severe crowding were improving, and their canines were erupting spontaneously once extractions were performed. Moreover, this theory was good support for the serial extraction planning used to be practiced in the past 50 years [22, 23].

There are various alterations thoroughly described in the literature that can affect the oral cavity, such as dental caries, abnormal teeth, enamel hypoplasia, supernumerary teeth, and dental agenesis. A syndromic patient with a complex picture showing conditions such as palatine fissures and severe hypodontia has to be treated using a multidisciplinary approach and, in particular, needs prosthetic rehabilitation for the restoration of missing dental elements in the arch [24–26].

This study aimed to estimate the occurrence, patterns, and sequelae of impacted canines and to evaluate the etiology, complications, and treatment options within the region of the Kurdistan, Iraq, population.

## 2. Materials and Methods

### 2.1. Study Setting

This study was conducted at the Orthodontic Department of Khanzad Teaching Center belonging to the General Directorate of Hawler, Ministry of Health, Kurdistan region, Iraq, during the years 2015–2021. All the patients who were seeking orthodontic treatment were examined after having a brief description of the study and obtaining informed consent to participate in the research. Only Iraqi patients in the age range of 18–50 years were included in the study.

### 2.2. Study Subjects

Case selection was performed clinically and radiographically by one researcher during the routine examination for admission to the orthodontic clinic. A total of 57800 patients were examined, and only 2239 patients were selected according to inclusion and exclusion criteria (males = 798, females = 1432). The patients' ages ranged from 18 to 50 (mean age 24.77 ± 6.06) years, which were distributed into three age groups: 18–30, 31–40, and 41–50 years old.

## 3. Examination

The selected cases were distributed to three orthodontists to be examined through regular diagnostic methods using a clinical examination sheet based on previous studies [27, 28]. The clinical examination involved visual inspection for canine bulges and teeth deviations and hand palpation for the presence of the canines within the buccal and palatal directions. An occlusal examination for articulation and teeth positioning was performed.

Radiographical investigations involved cone-beam computerized technology (CBCT machine, NewTom VG, QR s.r.l., Verona, Italy) which was used under the following settings: 15 3 15 cm field of view, 110 kV, and 1–20 mA (pulsed mode) with a resolution of 0.3 mm isotropic voxel and exposure time of 10 seconds.

**Figure 1:**
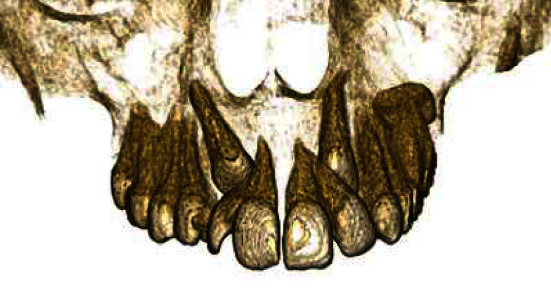
Bilateral impaction of maxillary canines.

The canine was deemed impacted when prevented from the eruption in the maxillary or mandibular arch beyond the date of the eruption, and the apex was completed, as shown in Figure 1.

The sheet was used to assess the impaction and consisted of five sections.

### 3.1. Patient's Demographic Data

It included the patient's age, gender, personal history, family history, medical history, and dental history.

### 3.2. Classification of the Impaction

**Figure 2:**
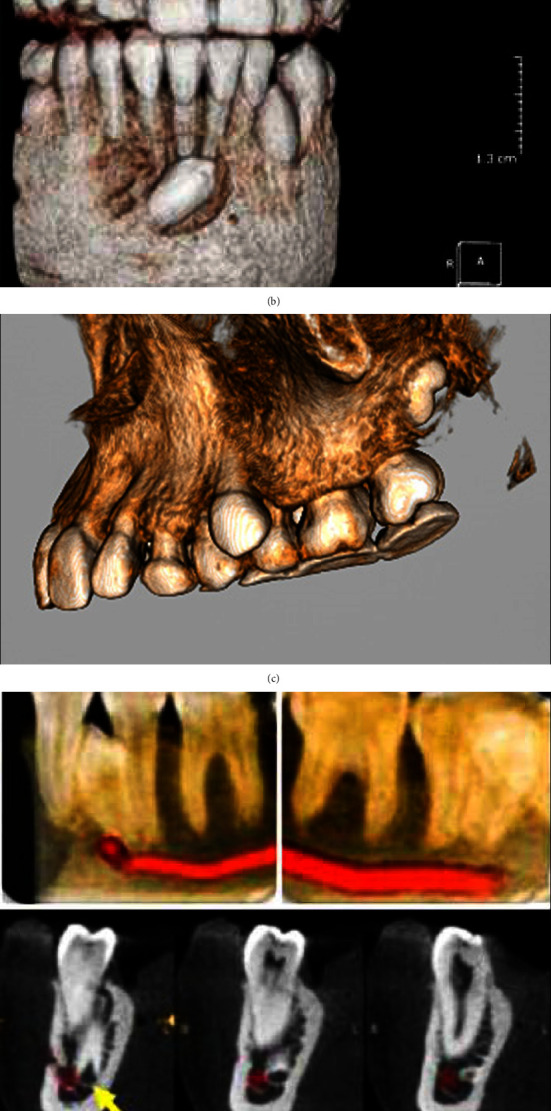
Mupparapu's classification using CBCT. (a) Type 1. (b) Type 2. (c) Type 3. (d) Type 4. (e) Type 5.

The position of maxillary and mandibular impacted canine teeth was classified according to Mupparapu's classification, as shown in Figure 2.

  Type 1: “Canine positioned mesioangular across the midline within the jaw bone, labial or lingual to anterior teeth, and the crown portion of the tooth was crossing the midline”
  Type 2: “Canine horizontally impacted near the inferior border of the mandible below the apices of the incisors”
  Type 3: “Canine was erupting either mesial or distal to the opposite canine”
  Type 4: “Canine horizontally impacted near the inferior border of the mandible below the apices of either premolars or molars on the opposite side”
  Type 5: “Canine was positioned vertically in the midline (the long axis of the tooth crossing the midline) irrespective of eruption status”

## 4. Etiology of Impaction

The etiology of the impaction is according to clinical and radiographic examination (supernumerary teeth, odontoma, pathological lesion “cysts,” delayed exfoliation of the deciduous canine, early trauma to the maxilla, cleft lip, and palate, ankylosis, displacement of the crypt, long path of eruption “hypoplasia, hypodontia or microdontia of the lateral incisor,” or “syndromes ” “cleidocranial dysplasia”) [24, 25].

## 5. Complications

Complications are associated with impacted canine during clinical and radiographic examination (root resorption of the adjacent permanent teeth, cystic changes “dentigerous cyst,” pain in the impacted tooth, swelling related to the impacted tooth, internal resorption, or migration of the neighboring teeth and loss of the arch length) [26].

## 6. Proposed Treatment

The proposed treatment for the selected cases is according to clinical examination and patient's preferences as observation, extraction, surgical exposure, and orthodontic alignment or surgical repositioning of the impacted tooth.

### 6.1. Statistical Analysis

Data were analyzed and tabulated using the Statistical Package for Social Sciences (SPSS, version 23). Numerical variables were presented in means and standard deviations. Categorical variables were presented in frequencies and proportions. The chi-square test was used to compare study groups at a significant level *p* < 0.05.

## 7. Results

The total number of patients who had been examined at the start of the study was 57800, who were seeking orthodontic treatment. By examination, 2230 patients (3.9%) had impacted canines (798 males and 1432 females).  Table 1 shows the demographic data of the selected sample. Most of the cases (69.9%) showed impaction in the upper jaw; whereas, only 30.1% of patients had impaction of the lower canines, with no statistical significance difference between males and females (Table 2).

Regarding the position of the impacted canines, the majority of impactions were mesioangular across the midline (type I = 54.8%), followed by 25% type II impaction; then, type III, type IV, and type V Mupparapu's classification were 10.4%, 4.8%, and 4.7%, respectively, with a statistically significant difference between males and females (Table 3).

The clinical and radiographical examination showed various etiological causes for canine impactions (Table 4). The delayed deciduous canine was the main feature in 45.9% of females and 33% of males (overall, 41% of patients had retained deciduous canines). Pathological cysts were found in 17.9% of females and 14.8% of males, while a long path of eruption was the second most common cause in males (26.6%) and the third for females (12.8% %). The etiology of impacted canines showed a highly statistically significant difference between the two genders (*p* < 0.001).

Canine impaction in examined patients showed different complications (Table 5 and Table 6). Pain was the most common complication in 67% of cases, followed by migration of neighboring teeth in 17.8% of cases. Swelling and cystic changes were less common at 7.7% and 6.1%, respectively. Finally, root resorption was found in only 1.4% of cases. The differences between genders and age groups were statistically significant (*p* < 0.001 and *p*=0.026, respectively).

The treatment methods of the examined cases based on the orthodontist's assessment of the case and patient's preferences are tabulated in Table 7 and Table 8, which showed no statistically significant difference between males and females, with 82% of total samples going for surgical exposure and orthodontic treatment. Moreover, 12.2% would be under observation, whereas 5.8% should undergo extraction of the impacted tooth. On the other hand, age groups showed a statistically significant difference (*p* < 0.001) as the majority of the 18–30 age group (91.3%) would go for surgery and orthodontics treatment, and more than half of the 30–40 years old age group would stay under observation. Finally, 50% of the 40–50 years age group would be treated with surgery and orthodontic treatment.

## 8. Discussion

Impacted canine cases were important to diagnose and handle in any orthodontic practice. In this study, the authors conducted a cross-sectional study to detect the location of impaction, the orientation of the impacted tooth, the etiology of impaction, the anticipated treatment methodology, and the complications. The study was formulated and conducted over the cases that attended the orthodontic clinic for consultation at the Orthodontic Department of Khanzad Teaching Center, within Kurdistan region, Iraq.

Case selection for the study was conducted by surveying the cases that arrived at the diagnosis clinic. If a case was suspected of an impacted canine, the case was guided into full records and investigations to confirm the diagnosis. The records included a questionnaire to record the history and chief complaints of the patients. That questionnaire investigated personal history as name, age, gender, job type, socioeconomic status, and demographical residency. Full clinical examination included inspection of the occlusion and oral cavity together with palpation of the arches and sulci, trying to locate the missing canines. The radiographical examination included cone-beam computerid tomography (CBCT) to locate the canines and arrange with the surgeon for the proper surgical approach.

Patients were categorized into age groups, the 18–30 years old group, the 30–40 years old group, and the 40–50 years old group. The data were collected and categorized according to Mupparapu's classification. The classification was based on the location and the orientation of the impacted canine, together with its relationship to other teeth. Data were plotted into tables and fed into the Statistical Package for Social Sciences (SPSS, version 23) program. Statistical analyses were into numerical variables as means and standard deviations. On the other hand, categorical data were presented as proportions. To compare the data between males and females, the chi-square test was used.

The total number of patients examined within the period of the study to represent the sample was 57800, and of those, 798 males and 1432 females showed impacted canines. That was represented by a ratio and prevalence of 3.9% of impacted canine cases in the sample. Those results came near to that found by Brin et al. [29] and Jacobs [30]. There was a significant association between the age of the patients and the prevalence of impacted canines in a certain arch. In all age groups, the chance of impaction was higher in the upper arch than that in the lower arch, as found by Jacobs [30] but different than what was found by McKay [31] and Power and Short [32]. That result was significant and evident. The younger the age group (18–30 years), the higher the chance of finding impacted canines. That might be due to a higher percentage seeking treatment at that age trying to improve their smile. The distribution between the males and females was not statistically significant; hence, we cannot assure if the prevalence had any association with gender [33–35].

Statistical examination of the data to link groups to Mupparapu's classification revealed a highly statistically significant relationship. Mupparapu's classification considered the position and orientation of the impacted canine [15]. The classification included five categories: type 1: canine was positioned mesioangular across the midline within the jaw bone, labial or lingual to anterior teeth, and the crown portion of the tooth was crossing the midline. Type 2: canine was horizontally impacted near the inferior border of the mandible below the apices of the incisors. Type 3: canine was erupting either mesial or distal to the opposite canine. Type 4: canine was horizontally impacted near the inferior border of the mandible below the apices of either premolars or molars on the opposite side. Type 5: canine was positioned vertically in the midline (the long axis of the tooth crossing the midline). By examining Table 2, one could detect that the prevalence was distributed according to the degree of classification. While, grade 1 was the most prevalent and grade 5 was the least to be detected. That was evident in all age groups except for the 30–40 years age group, where the prevalence of type 5 was more than type 4. Moreover, one could detect that the prevalence of each category was higher in the younger age group and decreased gradually when the age limit increased. These results came in coincidence with that found by Kadhim et al. [36].

Table 3 was important to detect the most and the least causative factor for the impaction incidence. We faced different factors regarding the different age groups. For the age group 18–30 years old, a retained deciduous canine was the most prevalent factor, while clefting of the palate was the least prevalent. But for the 30–40 age group, the retained deciduous canine was the most detected factor, and the odontoma was the least prevalent. While for the 40–50 age group, the long path of eruption was the most important factor, and some of the other factors, such as supernumeraries and odontomas, early traumas, and clefts, were not recorded in the sample. Those results came to differ from that found by Kamiloglu and Kelahmet [37]. While classifying the data according to gender, again, the retained deciduous canine was the most dominant factor in both groups. In females, the odontomas were the least prevalent, while for the males, clefts were the least evident factors.

In Table 4, the relationship between the complications and the different groups was evident. Pain in the impacted tooth was the most detectable complication in all age groups and gender divisions. On the other hand, root resorption was the least detectable complication in all groups, whether age or gender groups. For the 40–50 age group, cystic changes and swellings were not recorded at all, neither the root resorption. These results came near to that found by Jacobs [38] different from Kokich [16].

The decision of the treatment planning after examining the records and discussion with the patients was evident, as given in Table 5. For the age group 18–30, the most favorable choice was exposure and orthodontic alignment, while the least selected choice was to observe and follow up [39]. That presented the fact that young age was fully into ideal orthodontic treatment that corrects both the esthetics and function. The young-aged persons had time and were patients for more complex approaches, such as surgical exposure and orthodontic traction, that would consume two years of treatment duration. For the 30–40 age group, observation and follow-up were the preferred methods, while extraction was the least preferred method. One had to keep in mind that observation of canines did not mean that orthodontic treatment for leveling and alignment would not take place. Simply, the patients focused on the esthetics part of treatment and chose to neglect the impaction status and just perform leveling and alignment to improve the smile. But for the 40–50 age group, on the contrary [38], the highly selected method was to expose the canine surgically and correct its position through orthodontics, while the least selected method was to extract it. Those adult patients who were attending the orthodontic clinic on their own will mostly be referred from other interdisciplinary as implantologists and prosthodontists or TMD specialists, seeking functional improvement of the occlusion. That explained why in such an older-aged group, the choice mostly was to expose and correct. Cone-beam computed tomography (CBCT) can be considered the gold standard for radiologic diagnosis, providing detailed information about the length and width of the coronoid process and its relation to the zygomatic bone and arch [40].

Those results would explain the classical swinging demand of treatment between the esthetic and function. As we noticed from the study, the young-aged and teenagers were into full correction in both esthetics and function. On the contrary, young adults 30–40 years of age were more into esthetics and not much concerned about the function. On the other hand, adult patients above the age of 40s would focus more on occlusion and function to help solve interdisciplinary problems. The gender distribution revealed a nonsignificant statistical difference; hence, we cannot rely on the information depicted from their numbers [41]. It was critical to notice from this paper that the sample was not collected from the general population but from the orthodontic population who attended the orthodontic clinic for an orthodontic consultation. It was important to drive the attention that the results were to be applied to the orthodontic population only and not to be generalized to include the whole society.

## 9. Conclusions

The impacted canines were recorded in all age groups that attended the orthodontic clinic, with a 3.9% total prevalence and distribution between the two genders. The upper arch had more prevalence of impaction than the lower arch. The orientation and classification followed the hierarchy of Mupparapu's classification to a great degree. The retained deciduous canines were a real problem that caused many complications and needed a good educational session for the general dentist and the population for the younger age groups, while the long path of eruption was the main causative factor in the older age groups. The patients usually attended the clinic complaining of pain, and most of them were candidates for orthodontic traction. Most of the younger age groups went for exposure and traction, while the mid-aged groups were more into follow-ups and had a focus on esthetic concerns. The older-aged groups were into a full correction to help improve the occlusion for interdisciplinary case solutions.

## Figures and Tables

**Table 1:** Age distribution data for the selected cases.

		Gender					
		Females (*N*1 = 1432)		Males (*N*2 = 798)		Total (*N* = 2230)	
		No.	%	No.	%	No.	%
Age groups (years)	18–30 Y	1221	85.3	664	83.2	1885	84.5
	30–40 Y	206	14.4	121	15.2	327	14.7
	40–50 Y	5	0.3	13	1.6	18	0.8
	Total	1432	64.2	798	35.8	2230	100.0

**Table 2:** The relation between gender and site of impaction.

Location	Gender					
	Females (*N*1 = 1432)		Males (*N*2 = 789)		Total (*N* = 2230)	
	No.	%	No.	%	No.	%
Upper	993	69.3	566	70.9	1559	69.9
Lower	439	30.7	232	29.1	671	30.1
*χ* ^2^ (*p*)	0.611 (*p*=0.434)					

^*∗*^Chi-square test significant at *p* < 0.05.

**Table 3:** The relations between gender and Mupparapu's classification.

Type	Gender					
	Females (*N*1 = 1432)		Males (*N*2 = 789)		Total (*N* = 2230)	
	No.	%	No.	%	No.	%
Type I	796	55.6	427	53.5	1223	54.8
Type II	353	24.7	210	26.3	563	25.2
Type III	132	9.2	101	12.7	233	10.4
Type IV	84	5.9	22	2.8	106	4.8
Type V	67	4.7	38	4.8	105	4.7
*χ* ^2^ (*p*)	17.194 (0.002^*∗*^)					

^*∗*^Chi-square test significant at *p* < 0.05.

**Table 4:** The etiological factors of impacted canines for both genders.

Etiology	Gender					
	Females (*N*1 = 1432)		Males (*N*2 = 789)		Total (*N* = 2230)	
	No.	%	No.	%	No.	%
Supernumerary	52	3.6	45	5.6	97	4.3
Odontoma	13	0.9	20	2.5	33	1.5
Pathology (cyst)	256	17.9	118	14.8	374	16.8
Delayed Dec canine	657	45.9	263	33.0	920	41.3
Trauma	60	4.2	26	3.3	86	3.9
Cleft	25	1.7	8	1.0	33	1.5
Ankylosis	142	9.9	94	11.8	236	10.6
Crypt displacement	44	3.1	12	1.5	56	2.5
Long eruption path	183	12.8	212	26.6	395	17.7
*χ* ^2^ (*p*)	102.02 (*p* < 0.001)^*∗*^					

^∗^Chi-square test significant at *p* < 0.05.

**Table 5:** Complications of impacted canines for both genders.

Complications	Gender					
	Females (*N*1 = 1432)		Males (*N*2 = 789)		Total (*N* = 2230)	
	No.	%	No.	%	No.	%
Root resorption	29	2.0%	2	0.3%	31	1.4%
Cystic changes	63	4.4%	72	9.0%	135	6.1%
Pain	961	67.1%	535	67.0%	1496	67.1%
Swelling	132	9.2%	40	5.0%	172	7.7%
Migration of neighboring teeth	247	17.2%	149	18.7%	396	17.8%
*χ* ^2^ (*p*)	42.03 (*p* < 0.001)^*∗*^					

^*∗*^Chi-square test significant at *p* < 0.05.

**Table 6:** Complications of impacted canines for all age groups.

Complications	Age groups							
	18–30 years (*N*1 = 1885)		30–40 years (*N*2 = 327)		40–50 years (*N*3 = 18)		Total (*N* = 2230)	
	No.	%	No.	%	No.	%	No.	%
Root resorption	27	1.4	3	0.9	1	5.6	31	1.4
Cystic changes	119	6.3	16	4.9	0	0.0	135	6.1
Pain	1274	67.6	207	63.3	15	83.3	1496	67.1
Swelling	150	8.0	22	6.7	0	0.0	172	7.7
Migration of neighbors	315	16.7	79	24.2	2	11.1	396	17.8
*χ* ^2^ (*p*)	17.402 (*p*=0.026)^*∗*^							

^*∗*^Chi-square test significant at *p* < 0.05.

**Table 7:** Treatment of impacted canines in both genders.

Treatment	Gender					
	Females (*N*1 = 1432)		Males (*N*2 = 789)		Total (*N* = 2230)	
	No.	%	No.	%	No.	%
Observation	161	11.2	111	13.9	272	12.2
Extraction	91	6.4	39	4.9	130	5.8
Surgery and ortho	1180	82.4	648	81.2	1828	82.0
*χ* ^2^ (*p*)	4.971 (0.083)					

^*∗*^Chi-square test significant at *p* < 0.05.

**Table 8:** Treatment of impacted canines in different age groups.

Complications	Age groups							
	18–30 years (*N* = 1885)		30–40 years (*N*2 = 327)		40–50 years (*N*3 = 18)		Total (*N* = 2230)	
	No.	%	No.	%	No.	%	No.	%
Observation	90	4.8	174	53.2	8	44.4	272	12.2
Extraction	74	3.9	55	16.8	1	5.6	130	5.8
Surgery and ortho	1721	91.3	98	30.0	9	50.0	1828	82.0
*χ* ^2^ (*p*)	761.06 (*p* < 0.001)^*∗*^							

^*∗*^Chi-square test significant at *p* < 0.05.

## Data Availability

The data used to support the findings of this study are available from the corresponding author upon request.
